# CircNEIL3 mediates pyroptosis to influence lung adenocarcinoma radiotherapy by upregulating PIF1 through miR-1184 inhibition

**DOI:** 10.1038/s41419-022-04561-x

**Published:** 2022-02-21

**Authors:** Ting Zhang, Dong-Ming Wu, Peng-Wei Luo, Teng Liu, Rong Han, Shi-Hua Deng, Miao He, Yang-Yang Zhao, Ying Xu

**Affiliations:** 1 School of Clinical Medicine, Chengdu Medical College, Chengdu, Sichuan China; 2 The First Affiliated Hospital of Chengdu Medical College, Chengdu, Sichuan China

**Keywords:** Radiotherapy, Non-small-cell lung cancer

## Abstract

Circular RNAs (circRNAs) belong to an abundant category of non-coding RNAs that are stable and specific, and thus have great potential in cancer treatment. However, little is known about the role of circRNAs during radiotherapy in lung adenocarcinoma (LUAD). Here, we established the expression profiles of 1,875 dysregulated circRNAs in non-irradiated and irradiated A549 cells and identified circNEIL3 as a significantly downregulated circRNA in A549 cells treated with 0, 2, or 4 Gy of radiation, respectively. Functional assays demonstrated that circNEIL3 knockdown promoted radiation-induced cell pyroptosis, whereas circNEIL3 overexpression had the opposite effects. Importantly, the effects of circNEIL3 overexpression on inhibiting pyroptosis were reversed by PIF1 knockdown. Mechanistically, circNEIL3-mediated pyroptosis was achieved through directly binding to miR-1184 as a sponge, thereby releasing the inhibition of miR-1184 on PIF1, which ultimately induces DNA damage and triggers AIM2 inflammasome activation. In vivo, circNEIL3 knockdown significantly enhanced the efficacy of radiotherapy as evidenced by decreases in tumor volume and weight. Collectively, the circNEIL3/miR-1184/PIF1 axis that mediate pyroptosis induction may be a novel, promising therapeutic strategy for the clinical treatment of lung cancer.

## Introduction

Lung cancer remains the leading cause of cancer-related mortality accounting for 18% globally and 19% of all cancer-related deaths in China [1]. It is estimated that 85% of human lung cancers are non-small cell lung cancer, with lung adenocarcinoma (LUAD) being the predominant subtype, accounting for 50% of all cases [2]. Radiotherapy is widely used in the treatment of clinical tumors, related to lung cancer, prostate cancer, and kidney cancer, among others [3–6]. However, ionizing radiation can cause a range of opportunistic infections and systemic inflammation, with life-threatening tumor recurrence and metastasis still prevalent in patients [7, 8]. Therefore, further investigation is needed to gain a deeper understanding of lung cancer to provide new therapeutic targets for lung cancer radiotherapy.

Pyroptosis is a recently discovered form of programmed cell death, characterized by cell swelling and large bubbles emerging from the plasma membrane [9, 10]. Pyroptosis is divided into the caspase-1-activated classical and caspase-11/4/5-activated non-classical pathways. In the classical pathway, the assembled NLRP1, NAIP, NLRC4, AIM2, NLRP3, and pyrin proteins activate and cleave pro-caspase-1 to form active caspase-1, which then cleaves gasdermin D (GSDMD) to the N-terminal form (GSDMD-N) with its pore-forming activity, and ultimately releases inflammatory factors IL-1β and IL-18, causing inflammatory reactions [11, 12]. Conversely, in the non-classical pathway, lipopolysaccharide (LPS) can combine directly and activate caspase-11/4/5, which can then cleave GSDMD into GSDMD-N [11–13]. Recent studies have shown that pyroptosis plays an important role in the radiation process [14–16]. For example, NLRP3 inflammasome activation can mediate radiation-induced pyroptosis in bone marrow-derived macrophages [17]. Furthermore, mesenchymal stem cells can attenuate radiation-induced brain injury by inhibiting microglia pyroptosis [18]. However, little is known regarding the role of pyroptosis in lung cancer radiotherapy.

Circular RNAs (circRNAs), a category of non-coding RNAs characterized by covalently closed-loop structures without 5′−3′ polarity or a poly adenosine tail [19, 20], are abundant, conserved, stable in organisms, and resistant to RNase R treatment [21–23]. Since their first discovery in viruses in 1976, the functions of circRNAs in various human diseases have been reported, including cardiovascular diseases [24, 25], lupus nephritis [26], systemic neural diseases [27], and most cancers [22, 28]. Moreover, the radiosensitivity of bone marrow mesenchymal stem cells has been shown to be affected by circRNA_014511 binding to miR‑29b‑2‑5p [29]. Functionally, circRNAs are commonly used as competitive endogenous RNAs (ceRNAs) to eliminate the inhibitory effects of microRNA (miRNA) on target genes by acting as miRNA sponges.

Some important regulatory roles of circRNAs in terms of radiation have been reported, such as radiation-induced esophageal injury [30], the radiation sensitivity of esophageal cancer cells [31], and the radioresistance of HeLa cells [32]. However, the specific role and mechanism of circRNAs in lung cancer radiotherapy remain unclear. Therefore, we investigated the potential roles of circRNAs in the radiotherapy of lung cancer by establishing the expression profile of circRNAs and verified thousands of distinct circRNAs in non-irradiated and irradiated LUAD cells using high-throughput RNA sequencing (RNA-seq). Subsequently, we identified specific differentially expressed circRNAs and elucidated their roles in pyroptosis as potential therapeutic targets in lung cancer.

## Results

### circNEIL3 is significantly downregulated in irradiated LUAD cells

**Fig. 1:**
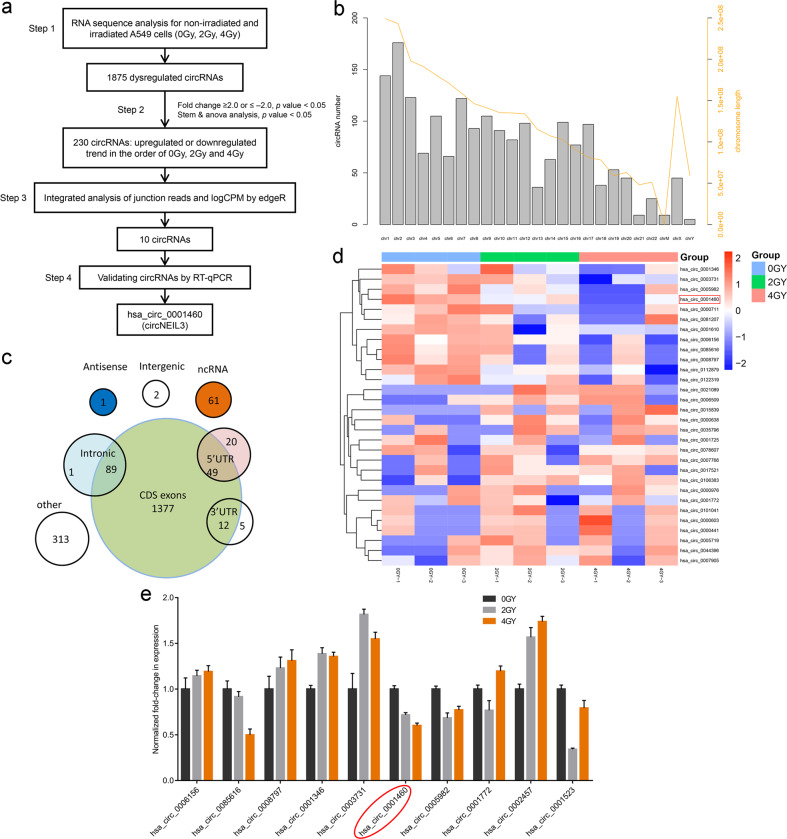
The circRNA expression profile workflow in the irradiated LUAD cells. **a** Flow chart for identifying and validating circRNAs in A549 cells. **b** Distribution map of circRNAs based on chromosome location. **c** Distribution map of circRNAs based on category. **d** Heatmap showing the top 15 most upregulated and top 15 most downregulated circRNAs in A549 cells treated with either 0, 2, or 4 Gy radiation, respectively. **e** Ten most representative circRNAs verified using RT-qPCR.

To investigate the roles of circRNAs in the radiotherapy of lung cancer, we performed RNA-seq and analyzed the expression profiles of circRNAs in non-irradiated and irradiated A549 lung cancer cells (sequences were deposited in the Gene Expression Omnibus under dataset code GSE124396; www.ncbi.nlm.nih.gov/geo). A total of 1875 dysregulated circRNAs were identified in irradiated A549 cells (Fig. 1a). The hierarchical clustering heatmap suggested that the expression levels of circRNAs were clustered and distinguishable (Supplementary Fig. S1a). The clonogenic survival assay showed that the 4 Gy dose of radiation treatment significantly reduced the proliferation ability of A549 cells (Supplementary Fig. S1b). In addition, the category and chromosomal localization of circRNAs were also identified (Fig. 1b, c). Based on the STEM and ANOVA analysis, 230 circRNAs showed an upregulated or downregulated trend in A549 cells treated with different doses (0, 2, or 4 Gy) of radiation (|fold change | ≥ 2.0, *P* < 0.05). Of these, the first 15 downregulated and 15 upregulated circRNAs are represented in Fig. 1d and listed in Table S1. Through integrated analysis of junction reads and logCPM by edgeR, we selected and validated 10 circRNAs using reverse transcription-quantitative polymerase chain reaction (RT-qPCR) (Fig. 1e and Table S2). The results showed that *hsa_circ_0001460* (termed circNEIL3) was the most significantly downregulated circRNA in the LUAD cells treated with either 0, 2, or 4 Gy of radiation, respectively (Fig. 1a, e).

### Characterization of circNEIL3 in LUAD cells

**Fig. 2:**
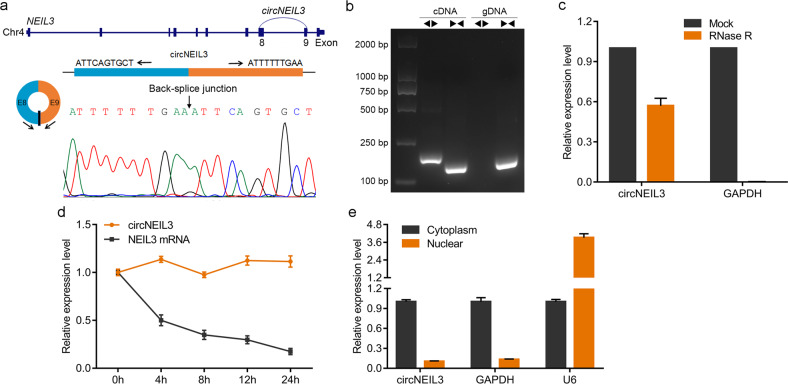
Characterization of circNEIL3 in LUAD cells. **a** Genomic location of circNEIL3. The back-spliced junction of circNEIL3 identified using Sanger sequencing. **b** PCR analysis for circNEIL3 in complementary DNA (cDNA) and genomic DNA (gDNA). **c** Relative levels of circNEIL3 and *GAPDH* detected using RT-qPCR after RNase R digestion, normalized to the value detected in the mock group. **d** RT-qPCR for the abundance of circNEIL3 and *NEIL3* in A549 cells treated with actinomycin D at the indicated time points. **e** Levels of circNEIL3 in the cytoplasmic and nuclear fractions of A549 cells.

We next evaluated the exon structure of circNEIL3, which is derived from exons 8 to 9 of the *NEIL3* gene with a length of 596 nucleotides located at chr4:178274462-178281831+. We also amplified the back-spliced junction of circNEIL3 using divergent primers, which was confirmed by Sanger sequencing (Fig. 2a). This sequence is consistent with the circBase database annotation (http://www.circbase.org/). Subsequently, PCR analysis showed that divergent primers only amplified a band in complementary DNA and not in genomic DNA (Fig. 2b). RT-qPCR after RNase R treatment showed that circNEIL3 was resistant to RNase R digestion compared to *GAPDH* (Fig. 2c). The stability of circNEIL3 was further analyzed in A549 cells treated with actinomycin D, an inhibitor of transcription, suggesting that circNEIL3 was more stable than *NEIL3* (Fig. 2d). To observe the cellular localization of circNEIL3, the nuclear and cytoplasmic circNEIL3 RNA was detected by RT-qPCR. As shown in Fig. 2e, circNEIL3 transcripts were preferentially located in the cytoplasm. These results demonstrated the characteristics of circNEIL3 as a circRNA, implying that its biological stability may be beneficial to its function.

### circNEIL3 deficiency is important for irradiation-induced pyroptosis in LUAD

**Fig. 3:**
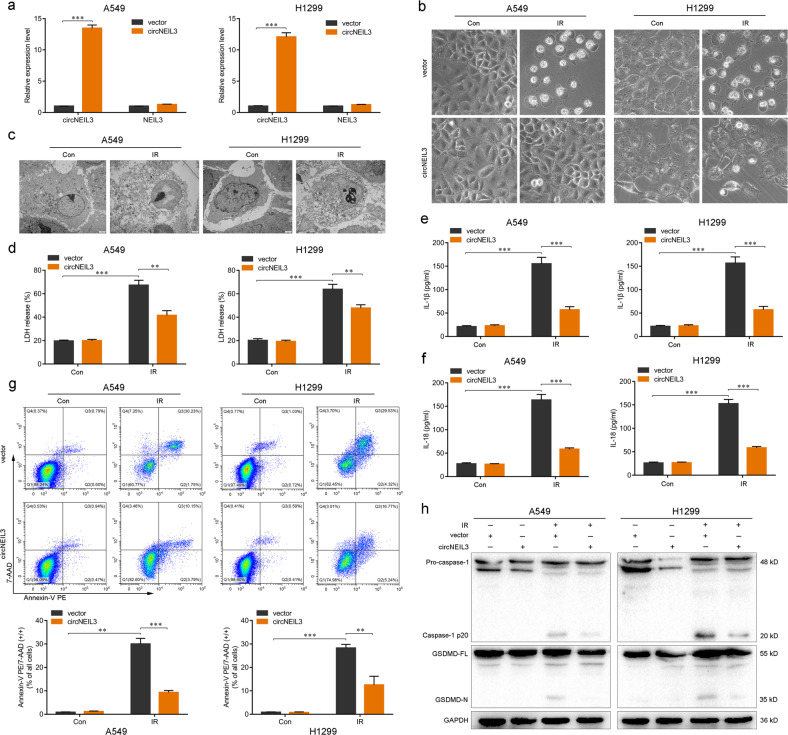
The importance of circNEIL3 expression in the irradiation-induced pyroptosis of LUAD cells. **a** Expression levels of circNEIL3 and NEIL3 in LUAD cells. **b** Representative images of LUAD cells treated with a 4-Gy dose of radiation after transfection for circNEIL3 overexpression. **c** Transmission electron microscopy images of LUAD cells 48 h after treated with 4 Gy dose of radiation. Scale bar 2 μm. **d**–**f** Enzyme-linked immunosorbent assays for quantifying the LDH, IL-1β, and IL-18 release of LUAD cells. **g** Percentage of Annexin V PE and 7-AAD double-positive cells in LUAD cells as detected using flow cytometry. **h** Analysis of pyroptosis markers (caspase-1, GSDMD-N) by western blotting in the indicated groups. **P < 0.01, ***P < 0.001, two-tailed Student’s *t*-test. *Con* control, *IR* irradiated.

To investigate the effect of circNEIL3 on LUAD radiotherapy, we utilized a small hairpin RNA lentiviral vector for stable knockdown of endogenous circNEIL3 in the A549 and H1299 cell lines. For ectopic overexpression of circNEIL3, exons 8–9 of *NEIL3* were cloned into the lentiviral vector. CircNEIL3 overexpression successfully upregulated circNEIL3 expression in both A549 and H1299 cells but had no effect on *NEIL3* mRNA expression (Fig. 3a). Similarly, circNEIL3 knockdown successfully downregulated circNEIL3 expression in LUAD cells, whereas *NEIL3* mRNA expression again showing no obvious change (Supplementary Fig. S2a). Morphologically, irradiation-treated A549 and H1299 cells exhibited large bubbles emerging from the plasma membrane and cell swelling, which highly resembled the characteristics of pyroptosis induced by the N-terminus of GSDMD [12], and circNEIL3 overexpression visibly inhibited this phenomenon (Fig. 3b). Conversely, stable knockdown of circNEIL3 remarkably increased the numbers of cell swelling and the formation of large bubbles (Supplementary Fig. S2b). The transmission electron microscopy also revealed multiple pores formed in the membranes of irradiation-treated A549 and H1299 cells (Fig. 3c).

Moreover, circNEIL3 overexpression significantly inhibited the release of lactate dehydrogenase (LDH) and the secretion of interleukin (IL)−1β and IL-18 in irradiated A549 and H1299 cells (Fig. 3d–f), whereas circNEIL3 knockdown led to a marked increase in the release of LDH and secretion of IL-1β and IL-18 (Supplementary Fig. S2c–e). Flow cytometry analysis showed fewer annexin V-PE and 7-AAD double-positive cells in circNEIL3-overexpressed A549 and H1299 cells treated with irradiation (Fig. 3g), whereas the opposite effects were observed for the circNEIL3 knockdown group (Supplementary Fig. S2f). Furthermore, western blotting showed that circNEIL3 overexpression suppressed the activity of caspase-1 to cleave GSDMD into GSDMD-N fragments with pore-forming activity (Fig. 3h), whereas, as expected, circNEIL3 knockdown had the opposite effects (Supplementary Fig. S2g). Taken together, these findings indicated that circNEIL3 deficiency plays a critical role in irradiation-induced pyroptosis in LUAD cells.

### circNEIL3 knockdown facilitates pyroptosis by targeting PIF1

**Fig. 4:**
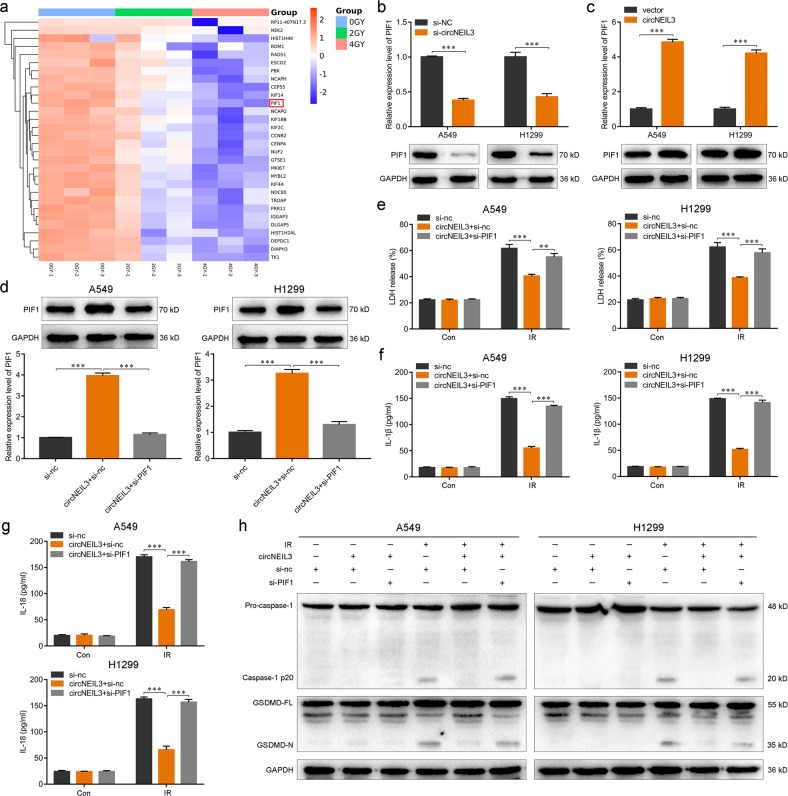
circNEIL3 facilitates pyroptosis by targeting PIF1. **a** Heatmap of the top 30 downregulated mRNAs in A549 cells treated with different doses (0, 2, or 4 Gy) of radiation. **b**, **c** Relative expression levels of PIF1 after circNEIL3 knockdown **b** or overexpression **c**. **d** Upregulation of PIF1 induced by circNEIL3 overexpression decreased by si-PIF1 treatment detected using both RT-qPCR and western blotting. **e**–**g** Enzyme-linked immunosorbent assays for LDH, IL-1β, and IL-18 release of cells in the indicated groups. **h** Upregulated expression of active-caspase-1 and GSDMD-N induced by circNEIL3 overexpression decreased by si-PIF1 treatment, detected using western blotting. **P < 0.01, ***P < 0.001, two-tailed Student’s *t*-test. Con, control; IR, irradiated.

To explore the molecular mechanism of circNEIL3 in regulating the pyroptosis of LUAD cells, we constructed and analyzed the ceRNA network for each circRNA using the miRanda and TargetScan tools (Supplementary Fig. S1c). Six predicted co-expressed mRNAs were found. Based on the hierarchical clustering heatmap of the first 30 mRNAs that showed a downward trend in the A549 cells treated with different doses (0, 2, or 4 Gy) of radiation, *PIF1* was the most obvious gene exhibiting a declining trend (Fig. 4a). Indeed, RT-qPCR and western blotting revealed that circNEIL3 knockdown significantly reduced the mRNA and protein levels of PIF1, and the opposite results were observed when circNEIL3 was overexpressed (Fig. 4b, c). Therefore, *PIF1* was considered as a major candidate target of circNEIL3.

Cell pyroptosis after knockdown of PIF1 was examined to further study the function of circNEIL3 on PIF1. Among the small interfering RNAs (siRNAs) tested, mRNA and protein levels of PIF1 were reduced by at least 60% with si-h-PIF1_001 (Supplementary Fig. S3); therefore, we utilized this siRNA for subsequent experiments. The PIF1 upregulation effect of circNEIL3 overexpression was clearly decreased following si-PIF1 treatment (Fig. 4d). Notably, PIF1 knockdown significantly attenuated the effects of circNEIL3 on reducing LDH, IL-1β, and IL-18 levels in irradiation-treated A549 and H1299 cells (Fig. 4e–g). Furthermore, the effects of circNEIL3 overexpression on the levels of active caspase-1 and GSDMD-N involved in pyroptosis were reversed by PIF1 knockdown (Fig. 4h). These results suggested that circNEIL3 potentially modulates LUAD pyroptosis by targeting PIF1.

### circNEIL3 regulates PIF1 expression via direct binding to miR-1184

**Fig. 5:**
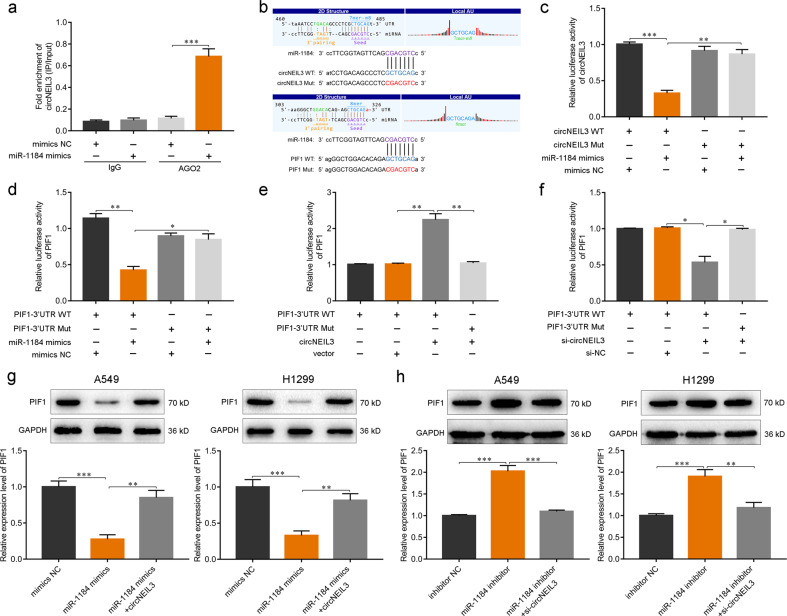
circNEIL3 regulates PIF1 expression via directly binding miR-1184. **a** RNA immunoprecipitation performed using AGO2 antibody in A549 cells transfected with miR-1184 mimics or negative control (NC) mimics, followed by the detection of circNEIL3 enrichment. **b** Wild-type (WT) and mutant (Mut) transcripts of circNEIL3 or the *PIF1* 3′-untranslated region (UTR) for luciferase reporters. **c**, **d** Dual luciferase reporter activity of circNEIL3 **c** and the PIF1 3′-UTR **d** in A549 cells co-transfected with either miR-1184 mimics or NC mimics. **e**, **f** Dual luciferase reporter activity of the *PIF1* 3′-UTR in A549 cells with circNEIL3 overexpression **e** or knockdown **f**. **g** Expression of PIF1 in LUAD cells transfected with miR-1184 mimics alone or co-transfected with circNEIL3. **h** Expression of PIF1 in LUAD cells transfected with miR-1184 inhibitor alone or co-transfected with si-circNEIL3. *P < 0.05, **P < 0.01, ***P < 0.001, two-tailed Student’s *t*-test.

circRNAs can eliminate the inhibitory effects of miRNAs on downstream targets by acting as miRNA sponges [22, 26, 33]. Given that circNEIL3 was found to be mainly located in the cytoplasm (Fig. 2e) and its interaction is established by the ceRNA network (Supplementary Fig. S1c), we next explored whether circNEIL3 could function as an miR-1184 sponge to regulate the expression of PIF1. AGO2 immunoprecipitation was performed to determine whether circNEIL3 served as a platform for AGO2 and miR-1184. Compared with IgG, circNEIL3 and miR-1184 were significantly enriched in the AGO2 pulldown complex (Fig. 5a). Subsequently, we constructed dual luciferase reporter assays containing wild-type and mutated putative binding sites for either circNEIL3 or *PIF1* transcripts to confirm that circNEIL3 and *PIF1* could be regulated by miR-1184 (Fig. 5b). The miR-1184 mimics significantly reduced the luciferase activity of the circNEIL3 wild-type reporter compared with that of either the control or mutated luciferase reporter (Fig. 5c). Similarly, the luciferase activity of the *PIF1* wild-type reporter was markedly decreased when transfected with miR-1184 mimics (Fig. 5d).

Moreover, circNEIL3 overexpression could further increase the luciferase activity of the *PIF1* wild-type reporter, whereas circNEIL3 knockdown reduced the activity level (Fig. 5e, f). Consistently, the miR-1184 mimics significantly reduced the mRNA and protein levels of PIF1 in A549 and H1299 cells, whereas circNEIL3 overexpression reversed these effects of miR-1184 on PIF1 expression (Fig. 5g). Conversely, circNEIL3 knockdown significantly eliminated the elevation of PIF1 expression caused by miR-1184 inhibitors (Fig. 5h). Taken together, these results demonstrated that circNEIL3 could directly bind to miR-1184 as a sponge to regulate the expression of PIF1.

### The circNEIL3/miR-1184/PIF1 axis mediates pyroptosis via the DNA damage pathway

**Fig. 6:**
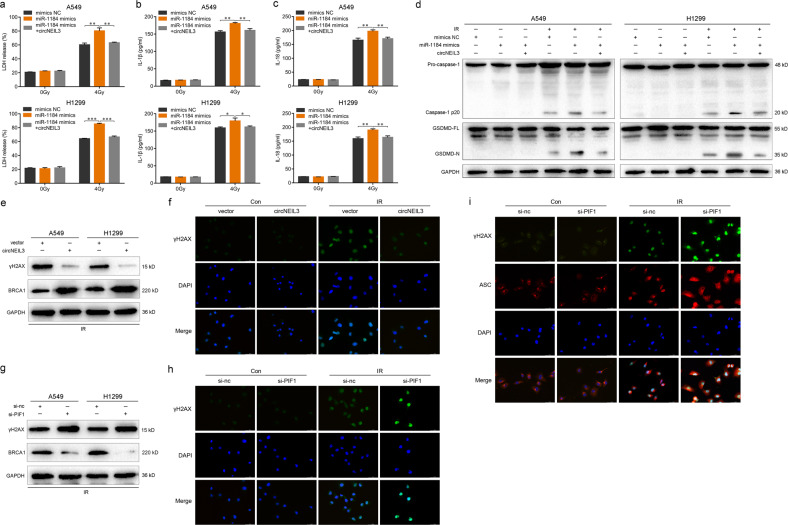
The circNEIL3/miR-1184/PIF1 axis mediates pyroptosis via the DNA damage repair pathway. **a**–**c** Enzyme-linked immunosorbent assays detecting the release of LDH, IL-1β, and IL-18 in LUAD cells transfected with miR-1184 mimics alone or co-transfected with circNEIL3. **d** Western blot of the expression of active-caspase-1 and GSDMD-N in the indicated groups. **e** Expression levels of γH2AX and BRCA1 in irradiated LUAD cells with circNEIL3 overexpression. **f** Immunofluorescence staining of γH2AX in A549 cells with or without circNEIL3 overexpression. **g** Expression levels of γH2AX and BRCA1 in irradiated LUAD cells with PIF1 knockdown. **h** Immunofluorescence staining of γH2AX in A549 cells treated with si-nc or si-PIF1. **i** Immunofluorescence co-staining of γH2AX and ASC in A549 cells treated with si-nc or si-PIF1. Scale bar, 50 μm. *P < 0.05, **P < 0.01, ***P < 0.001, two-tailed Student’s *t*-test. Con, control; IR, irradia*t*ed.

Based on the results described above, we assessed the regulatory role of the circNEIL3/miR-1184/PIF1 axis on the occurrence of cell pyroptosis. The increase in the release of LDH, IL-1β, and IL-18 induced by miR-1184 mimics was remarkably suppressed by circNEIL3 overexpression in irradiation-treated A549 and H1299 cells (Fig. 6a–c). The levels of active caspase-1 and GSDMD-N were evidently increased by miR-1184 mimics, whereas circNEIL3 overexpression abolished this effect (Fig. 6d). These results suggested that pyroptosis mediated by the circNEIL3/miR-1184/PIF1 axis is important for the radiotherapy of LUAD.

Previous studies have shown that the host *NEIL3* gene of circNEIL3 is a DNA glycosylase, which functions to remove DNA oxidative damage and modifies base excision repair [34, 35]. PIF1 helicase can affect the DNA replication of telomeres, ribosomes, and mitochondria; prevents the inappropriate addition of telomeres during DNA double-strand breaks; and reduces DNA damage [36–38]. Therefore, we next analyzed the γH2AX, a key marker of DNA damage.

After irradiation treatment, circNEIL3 overexpression significantly decreased γH2AX but increased BRCA1 protein levels (Fig. 6e). Whereas PIF1 knockdown promoted γH2AX, it inhibited BRCA1 protein levels compared to the control group (Fig. 6g). In parallel, immunofluorescence assay showed that the expression of γH2AX was reduced by circNEIL3 overexpression (Fig. 6f) but was increased by PIF1 knockdown (Fig. 6h). As DNA damage can activate the AIM2 inflammasome, thereby inducing cytokine production and GSDMD-mediated pyroptosis [39–41], we further detected the localization of ASC specks that co-stain with γH2AX. In A549 cells exposed to radiation following PIF1 knockdown, many of the ASC specks were detected in the nucleus of the DNA-damaged cells co-stained with γH2AX (Fig. 6i). Overall, these findings suggested that the circNEIL3/miR-1184/PIF1 axis mediates cell pyroptosis through the DNA damage pathway.

### circNEIL3 knockdown promotes radiation efficiency in vivo

**Fig. 7:**
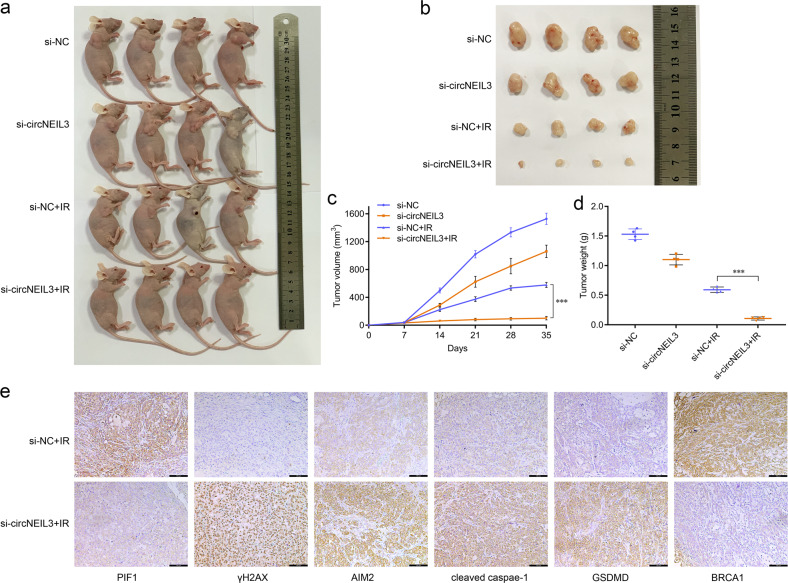
circNEIL3 knockdown promotes the radiation efficiency in vivo. **a** Images of nude mice with subcutaneous xenografts in the indicated groups. **b** Xenograft tumors isolated from sacrificed mice with or without radiation treatment (0 or 4 Gy/time/week) at the end of the experiment. **c** Volumes of subcutaneous xenograft tumors. **d** Average weights of xenograft tumors in the indicated groups. **e** Immunohistochemistry staining of DNA damage and pyroptosis biomarkers in subcutaneous tumors of mice injected with si-NC+IR vs si-circNEIL3+IR (scale bar, 100 μm).

To further explore the therapeutic potential of circNEIL3 in vivo, we established a subcutaneous tumor model in nude mice using A549 cells with or without circNEIL3 knockdown (Fig. 7a). Tumor xenograft analysis indicated that circNEIL3 knockdown efficiently enhanced the sensitivity of xenograft tumors to radiation treatment, and reduced the size and weight of the xenograft tumors (Fig. 7b–d). Immunohistochemistry analysis of tumor xenograft samples suggested that in the radiation-treated group, circNEIL3 knockdown elevated the expression of γH2AX, AIM2, cleaved caspase-1, and GSDMD, but reduced the levels of PIF1 and BRCA1 (Fig. 7e).

## Discussion

The circRNA expression profile influences the occurrence and progression of cancer. Compared with linear parental genes, circRNAs are expressed more widely, stably, and specifically in cells, tissues, and body fluids [21, 42, 43], and thus have greater potential as targets for cancer treatment. However, the role of circRNAs in the process of pyroptotic cell death during LUAD radiotherapy remains unclear. In this study, we established, the first (to the best of our knowledge) expression profile of circRNAs in non‑irradiated and irradiated LUAD cells through RNA-seq. Subsequently, we identified circNEIL3 as a significantly downregulated circRNA in the LUAD cells treated with 0, 2, or 4 Gy of radiation. We then performed gain-of-function and loss-of-function experiments of circNEIL3 in LUAD cells, showing that circNEIL3 knockdown promoted cell pyroptosis, while circNEIL3 overexpression had the opposite effects, indicating that circNEIL3 was associated with the pyroptosis of LUAD cells treated with radiation.

The ceRNA hypothesis proposes that circRNAs eliminate the inhibitory effects of miRNAs on mRNA by acting as miRNA sponges, constructing a complex post-transcriptional regulatory network [19, 22, 44]. Comprehensive analysis of the ceRNA network and heatmap of the top 30 downregulated mRNAs revealed that *PIF1* might be the downstream target of circNEIL3. The experimental findings showed that circNEIL3 knockdown significantly reduced mRNA and protein levels of PIF1, whereas circNEIL3 overexpression had the opposite effects. The effects of circNEIL3 overexpression on inhibiting pyroptosis were also reversed by PIF1 knockdown in irradiation-treated LUAD cells.

Previous studies have reported that circRNAs and mRNAs could share miRNA response elements, competing for the binding to miRNAs, and regulating each other’s expression [21–23]. We found that miR-1184 markedly reduced the luciferase activity of the circNEIL3 luciferase reporter, and this miRNA was subsequently verified as the binding target of circNEIL3. In addition, PIF1 was identified as the direct target of miR-1184, and circNEIL3 significantly attenuated the effects of miR-1184 on PIF1. These results indicate that circNEIL3 may function as a ceRNA to regulate PIF1 by binding to miR-1184 as a sponge in LUAD.

The extent of cell pyroptosis was increased by miR-1184 mimics, whereas circNEIL3 overexpression distinctly abolished the effects of miR-1184. The host gene *NEIL3* of circNEIL3 plays an important role in eliminating DNA oxidative damage and base excision repair [34, 35, 45]. PIF1 helicase functions also affect DNA replication and DNA damage [36]. Therefore, we speculated that circNEIL3 could also affect DNA damage repair. Interestingly, the radiation-induced expression of γH2AX, a marker of DNA damage, was significantly weakened by circNEIL3 overexpression but was enhanced by PIF1 knockdown. In this study, we demonstrated that ASC specks co-stained with γH2AX in cells exposed to radiation after PIF1 knockdown, which suggests that the circNEIL3/miR-1184/PIF1 axis mediates cell pyroptosis induced by radiation through regulation of the DNA damage repair pathway.

To determine the effect of circNEIL3 on lung cancer radiotherapy in vivo, we established an A549 subcutaneous tumor model and showed that circNEIL3 knockdown can enhance radiation efficiency, resulting in a reduced size and weight of xenograft tumors accompanied by a higher expression of DNA damage and pyroptosis markers. Our study lacks validation with clinical specimens since it is difficult to obtain lung cancer tissue specimens following radiotherapy. Nonetheless, our results confirm the role of the circNEIL3/miR-1184/PIF1 axis in both tumor cells and subcutaneous tumors in mediating pyroptosis to regulate LUAD radiotherapy by regulating the DNA damage repair pathway.

**Fig. 8:**
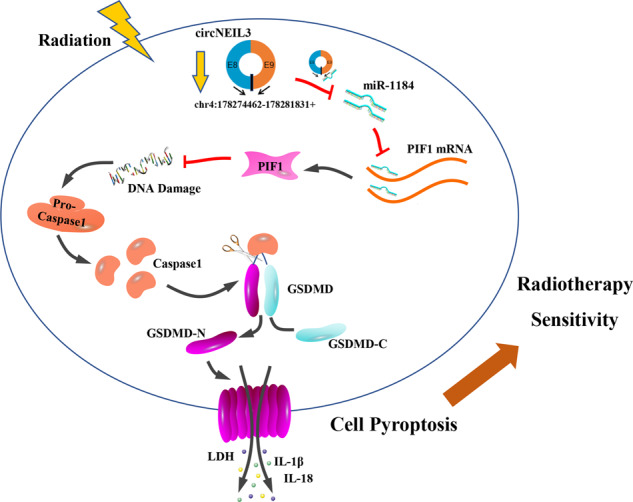
Diagram of the proposed mechanism and function of circNEIL3 in lung adenocarcinoma radiotherapy. circNEIL3 exerts its function as a ceRNA by directly binding to miR-1184, thereby abrogating the endogenous inhibitory effect of miR-1184 on the target gene PIF1, and inducing significant DNA damage, which in turn triggers AIM2 inflammasome activation to induce pyroptosis, ultimately affecting survival in lung cancer radiotherapy.

Collectively, this study demonstrates that circNEIL3 exerts its function as a ceRNA by directly binding to miR-1184, thereby abrogating the endogenous inhibitory effect of miR-1184 on the target gene *PIF1*, and inducing significant DNA damage, which in turn triggers AIM2 inflammasome activation to induce pyroptosis, ultimately affecting survival in lung cancer radiotherapy (Fig. 8). These results suggest that the circNEIL3/miR-1184/PIF1 axis mediates cell pyroptosis by regulating the DNA damage repair pathway. Therefore, circNEIL3-related pyroptosis induction may serve as a potential therapeutic target to enhance radiotherapy in lung cancer.

## Materials and methods

### Cell lines and cultures

The human LUAD cell lines A549 and NCI‑H1299 were purchased from The American Type Culture Collection and were authenticated by STR DNA profiling analysis. The LUAD cell lines were maintained in RPMI-1640 medium supplemented with 10% fetal bovine serum, 10 mM l-glutamine, and 5 mg/ml penicillin/streptomycin at 37 °C with 5% CO_2_. All media and supplements were purchased from Invitrogen (Carlsbad, CA, USA).

### Irradiation treatment

A549 cells (1 × 10^4^ cells/well) were seeded in 96‑well plates. After incubation for 24 h, the cells were exposed to various doses (0, 2, or 4 Gy) of radiation using the X‑RAD 160‑225 instrument (42 cm; 225 kV/s; 12.4 mA; 2.0 Gy/min; filter, 2 mm aluminum; Precision X‑Ray, North Branford, CT, USA) [46]. Based on our previous study [47], we performed the optimal radiosensitivity assessment after 48 h of radiation treatment. The irradiated LUAD cell lines refer to the adherent cells in the petri dish 48 h after 4 Gy dose of radiation treatment.

### Total RNA extraction and RT-qPCR

Total RNA was extracted using Total RNA Extraction Kit (Solarbio, Beijing, China), according to the manufacturer’s instructions, which was reverse-transcribed using iScript cDNA Synthesis Kit (Bio-Rad, Hercules, CA, USA). qPCR was then performed using a CFX96 Real-time System (Bio-Rad) with SYBR Green Supermix (Bio-Rad). Both procedures were performed according to the manufacturer’s instructions. The β-actin was used to be the internal controls of RT-qPCR analysis. The sequences of the primers used in this study are listed in Table S3.

### circRNA sequencing analysis

RNA libraries were constructed by CloudSeq Pte Ltd (Shanghai, China). circRNA-seq analysis was also performed by CloudSeq Pte Ltd. with a Bioanalyzer 2100 (Agilent, Santa Clara, CA) and sequenced by HiSeq 2000 (Illumina, San Diego, CA) on a 100 bp paired-end run [48]. The RNA-seq data were deposited in the Gene Expression Omnibus database (accession code: GSE124396).

### Cell viability assay

Cell viability was assessed by clonogenic survival assay. When the cell density reached 70%, cells were exposed to various doses (0, 2, or 4 Gy) of radiation. After 48 h, trypsin digestion was performed, cells were counted and re-seeded at 500 cells per well in a 6-well plate (Corning, Corning, NY, USA). Cells were cultured for 10 days with medium changes every 3 days. Colonies were washed with PBS, fixed in methanol, and stained with crystal violet. The data were expressed as percent survival relative to the control: ((treated count)/(average ctrl count)) × 100%.

### RNase R treatment

Total RNA (2 μg) was incubated at 37 °C for 30 min with or without 3 U/μg RNase R (Epicentre Technologies, Thane, Maharashtra, India), and subsequently purified using RNeasy MinElute Cleaning Kit (Qiagen, Hilden, Germany). All of the products obtained were used as substrates for complementary DNA synthesis and were analyzed by RT-qPCR.

### Actinomycin D assay

A549 cells were exposed to 2 μg/ml actinomycin D (Sigma-Aldrich, St. Louis, MO, USA) at various time points (0, 4, 8, 12, and 24 h). Cells were harvested and total RNA was extracted. The stability of circNEIL3 and *NEIL3* mRNA was analyzed using RT-qPCR.

### Cytoplasm and nuclear localization

The cytoplasm and nuclear fractions were separated and isolated from cells using NE-PER Extraction Kit (Thermo Scientific, Waltham, MA, USA) according to the manufacturer’s protocol. Cytoplasmic and nuclear RNAs were then converted to complementary DNAs and detected by RT-qPCR.

### Vector construction and cell transfection

To overexpress circNEIL3 in LUAD cells, GM-7183: GPLVX-circRNA _Mini-GFP-Puro was used as the lentiviral vector and 1E8TU was used as the control virus. For circNEIL3 knockdown, siRNAs targeting the si-h-coding sequences were designed and inserted into a 2494-PGMLV-SC5 RNAi lentiviral vector. Both of the constructions of circNEIL3 overexpression and knockdown were performed by Genomeditech (Shanghai, China). Overexpression and knockdown efficiencies were evaluated by RT-qPCR. Cells were transfected using Lipofectamine 3000 (Invitrogen) and harvested after 48 h for further experiments.

### PIF1 knockdown

PIF1 was silenced in LUAD cells with an siRNA Kit (RiboBio, Guangzhou, China), according to the manufacturer’s instructions. The target sequences were as follows: si-h-PIF1_001, 5′-GGTTGGTCATTGACGAGAT-3′; si-h-PIF1_002, 5′-GAGGCAGACCTGTTTGACA-3′; and si-h-PIF1_003, 5′-GGGAAGTCATATCTGCTAA-3′. The corresponding negative control was also purchased from RiboBio. Knockdown efficiency was assessed by RT-qPCR and western blotting.

### LDH, IL-1β, and IL-18 release assay

LDH release was detected using LDH Cytotoxicity Assay Kit (Beyotime). The levels of IL-1β and IL-18 were measured with enzyme-linked immunosorbent assays using QuantiCyto IL-1β and IL-18 ELISA Kit, respectively (Enzyme-linked Biotechnology, Shanghai, China), according to the manufacturer’s instructions. The absorbance value was measured at 450 nm. Each experiment was repeated three times.

### Flow cytometry

Flow cytometry was used to measure pyroptosis using Annexin V-PE/7-AAD Detection Kit (KeyGEN, Jiangsu, China), according to the manufacturer’s instructions. The cells were analyzed using a flow cytometer (FACSCalibur, Becton-Dickinson, Franklin Lakes, NJ, USA).

### Western blot

The specific steps of the western blot assay were performed, as previously described [22, 33] with the following antibodies: anti-Caspase-1 (ab179515), anti-GSDMD (ab219800), anti-GSDMD-N (ab215203), anti-ASC (ab180799) and anti-AIM2 (ab93015) (Abcam, Cambridge, UK); anti-γH2AX (9718) (CST, Danvers, MA, USA); anti-PIF1 (19006-1-AP), anti-BRCA1 (22362-1-AP), anti-GAPDH (60004-1-Ig), and the secondary antibodies (Proteintech, Wuhan, China). Blots were conjugated with the chemiluminescent horseradish peroxidase substrate (Millipore), visualized, and quantified by Quantity 5.2 (Bio-Rad) according to the manufacturers’ instructions.

### RNA immunoprecipitation

RNA immunoprecipitation experiments were performed using Magna RIP RNA-Binding Protein Immunoprecipitation Kit (Millipore) according to the manufacturer’s protocols. AGO2 antibody was used for the RNA immunoprecipitation assay (Cell Signaling Technology). Co-precipitated RNA was detected by RT-qPCR.

### Dual-luciferase reporter assay

For the luciferase reporter vector, the sequence of circNEIL3 and the *PIF1* 3′ untranslated region was cloned downstream of the pGL3-promoter. For the mutant version, the sequence fragment of circNEIL3 (478–484: GCTGCAG), the binding site for miR-1184, was changed to CGACGTC with pmiR-RB-Report^TM^ vector (RiboBio). Similarly, the sequence fragment of *PIF1* (319–325: GCTGCAG) was changed to CGACGTC with pmiR-RB-Report^TM^ vector. The constructed and identified wild-type and mutant circNEIL3 and *PIF1* forms were verified by RiboBio. The specific steps of the dual luciferase reporter assay were performed as described previously [22]. The luciferase activity was measured using a dual luciferase reporter assay system (Promega, Madison, WI, USA). The luciferase values were normalized to the corresponding *Renilla* luciferase values, and then the fold changes were calculated.

### Immunofluorescence

The immunofluorescence assay was performed according to previous reports [7, 23]. Immunofluorescence images were obtained using an Olympus BX51 microscope (Olympus, Tokyo, Japan) equipped with various objectives and a DP50 camera. Images were processed using DPC controller software (Olympus).

### Mouse xenograft model

All xenograft experiments were performed in accordance with the guidelines of the Laboratory Animal Ethical Committee at Chengdu Medical College. All experimental protocols were approved by the Laboratory Animal Ethical Committee at Chengdu Medical College. A subcutaneous tumorigenic model was established to study the role of circNEIL3 on the efficacy of LUAD radiotherapy in vivo. BALB/c-Female nude mice (4–5 weeks of age, 14–16 g) were purchased from Dossy Experimental Animals Co., Ltd (Chengdu, China). Before performing the experiment, mice were randomly assigned to four treatment groups (si-NC, si-circNEIL3, si-NC + IR and si-circNEIL3+IR), with four mice in each group. A549 cells with or without circNEIL3 knockdown (1 × 10^6^) were suspended in 100 μl serum-free RPMI-1640 medium and injected subcutaneously into the axilla of each nude mouse. Seven days after cell implantation, when the tumor volume was approximately 30–40 mm^3^, each nude mice in the IR groups was exposed to radiation treatment under anesthesia using an X-RAD 160-225 instrument (Precision XRay, Inc., Branford, CT, USA; filter: 2 mm AI; 42 cm, 225 kV/s, 12.4 mA, 2.0 Gy/min) (4 Gy dose/each time per mice per week). Individual cylindrical lead cover shields were used so that only the subcutaneous tumor in the axilla was exposed to radiation. Vernier calipers were used to measure the tumor growth every 7 days, according to the formula *V* = (a × b^2^)/2 to detect the tumor volume, where a and b are the maximum and minimum diameter in millimeters, respectively. After four weeks, the mice were sacrificed and weighed immediately after dissection.

### Immunohistochemistry

Immunohistochemistry was performed as previously reported [23]. The images were obtained using an Olympus BX51 microscope (Olympus, Tokyo, Japan) equipped with various objectives and a DP50 camera. Images were processed using DPC controller software (Olympus).

### Statistical analysis

All experiments in this study were independently performed with at least three biological replicates. Data analysis was performed with the statistical program GraphPad Prism 7.0 (GraphPad Software, San Diego, CA, USA). The results are presented as the mean ± standard deviation unless otherwise indicated. Statistical analyses were performed using two-tailed Student’s t-tests to compare the significance of differences between two experimental groups. A value of *P* < 0.05 was considered statistically significant.

## Supplementary information

aj-checklist

Supplementary figure and table legends

Table S1. The top 30 dysregulated circRNAs in A549 cells treated with different doses of radiation.

Table S2. The top 10 meaningful circRNAs based on the junction reads and logCPM values by edgeR.

Table S3. Primer sequences for RT-qPCR used in this study.

Figure S1. Bioinformatics analysis of circRNAs in LUAD cells.

Figure S2. circNEIL3 knockdown can promote irradiation-induced pyroptosis in LUAD cells.

Figure S3. Knockdown efficiency of PIF1 detected by RT-qPCR and western blot.

## Data Availability

The RNA-seq data were deposited in the GEO database (accession code: GSE124396).
