# Supplementary material for: CircNEIL3 mediates pyroptosis to influence lung adenocarcinoma radiotherapy by upregulating PIF1 through miR-1184 inhibition
Source: Cell Death Dis. 2022 Feb 21;13(2):167. doi: 10.1038/s41419-022-04561-x (PMC8861163; doi:10.1038/s41419-022-04561-x)
Supplement: Supplementary file 2 — Supplementary figure and table legends [file 41419_2022_4561_MOESM2_ESM.docx]

**Supplementary Information**

**Tables:**

**Table S1.** The top 30 dysregulated circRNAs in A549 cells treated with different doses of radiation.

**Table S2.** The top 10 meaningful circRNAs based on the junction reads and logCPM values by edgeR.

**Table S3.** Primer sequences for RT-qPCR used in this study.

**Figures:**

**Fig. S1** Bioinformatics analysis of circRNAs in LUAD cells. **a** Heatmap showing 1875 differentially expressed circRNAs in A549 cells treated with different doses (0, 2, 4 Gy) of radiation. **b** The clonogenic survival assay showing the responses of A549 cells to different doses (0, 2, 4 Gy) of radiation. **c** The circRNA-miRNA-mRNA network for the top 10 most meaningful circRNAs based on the junction reads and logCPM values by edgeR. **P* < 0.05, ****P* < 0.001, two-tailed Student’s t-test.

**Fig. S2** circNEIL3 knockdown can promote irradiation-induced pyroptosis in LUAD cells. **a** Expression levels of circNEIL3 and NEIL3 in LUAD cells treated with circNEIL3 siRNA. **b** Representative images of LUAD cells in the indicated groups. **c–e** Enzyme-linked immunosorbent assays for LDH, IL-1β, and IL-18 release of LUAD cells. **f** Percentage of Annexin V PE and 7-AAD double-positive cells in LUAD cells as detected by flow cytometry. **g** Analysis of pyroptosis markers (caspase-1, GSDMD-N) by western blotting in LUAD cells with and without circNEIL3 knockdown. **P* < 0.05, ***P* < 0.01, ****P* < 0.001, two-tailed Student’s t-test. Con, control; IR, irradiated.

**Fig. S3** Knockdown efficiency of PIF1 detected by RT-qPCR and western blot. ****P* < 0.001.
