# Supplementary material for: CircNEIL3 mediates pyroptosis to influence lung adenocarcinoma radiotherapy by upregulating PIF1 through miR-1184 inhibition
Source: Cell Death Dis. 2022 Feb 21;13(2):167. doi: 10.1038/s41419-022-04561-x (PMC8861163; doi:10.1038/s41419-022-04561-x)
Supplement: Supplementary file 3 — Table S1. The top 30 dysregulated circRNAs in A549 cells treated with different doses of radiation. [file 41419_2022_4561_MOESM3_ESM.docx]

| **circRNA** | **Gene sample** | | | **logCPM by edgeR** | | | | | | | | |
| --- | --- | --- | --- | --- | --- | --- | --- | --- | --- | --- | --- | --- |
| **downregulation (15)** | |  | 0Gy-1 | | 0Gy-2 | 0Gy-3 | 2Gy-1 | 2Gy-2 | 2Gy-3 | 4Gy-1 | 4Gy-2 | 4Gy-3 |
| hsa_circ_0112879 | | ZNF124 | 12.20 | | 12.83 | 12.70 | 10.73 | 11.31 | 11.33 | 10.18 | 11.06 | 7.66 |
| hsa_circ_0000711 | | NFATC3 | 11.26 | | 11.56 | 12.05 | 11.95 | 10.78 | 10.80 | 10.18 | 10.19 | 11.39 |
| hsa_circ_0008797 | | GSK3B | 11.26 | | 9.83 | 10.82 | 9.90 | 7.66 | 9.95 | 7.66 | 10.19 | 7.66 |
| hsa_circ_0122319 | | PLOD2 | 9.89 | | 11.87 | 12.30 | 9.90 | 9.94 | 11.33 | 7.66 | 11.06 | 7.66 |
| hsa_circ_0001346 | | RNF13 | 13.17 | | 12.52 | 11.74 | 13.49 | 11.70 | 12.02 | 11.04 | 11.06 | 12.34 |
| hsa_circ_0006156 | | FNDC3B | 11.26 | | 9.83 | 10.82 | 10.73 | 9.94 | 9.95 | 7.66 | 10.19 | 7.66 |
| hsa_circ_0001460 | | NEIL3 | 12.41 | | 11.56 | 11.35 | 9.90 | 9.94 | 10.80 | 7.66 | 7.66 | 10.01 |
| hsa_circ_0001610 | | TNFRSF21 | 11.64 | | 11.87 | 11.74 | 11.65 | 7.66 | 10.80 | 10.18 | 10.19 | 10.01 |
| hsa_circ_0078607 | | SLC22A3 | 12.60 | | 12.33 | 7.66 | 11.65 | 11.70 | 7.66 | 11.04 | 11.06 | 11.39 |
| hsa_circ_0001725 | | LMTK2 | 11.26 | | 12.12 | 9.98 | 10.73 | 11.70 | 9.95 | 10.18 | 11.99 | 10.01 |
| hsa_circ_0081207 | | LMTK2 | 9.89 | | 11.18 | 10.82 | 9.90 | 7.66 | 9.95 | 7.66 | 7.66 | 11.78 |
| hsa_circ_0001772 | | RBM33 | 11.26 | | 9.83 | 11.74 | 11.26 | 10.78 | 7.66 | 10.18 | 11.60 | 10.01 |
| hsa_circ_0085616 | | ASAP1 | 11.26 | | 9.83 | 10.82 | 10.73 | 7.66 | 9.95 | 7.66 | 10.19 | 7.66 |
| hsa_circ_0005982 | | PTK2 | 11.64 | | 10.66 | 12.30 | 9.90 | 10.78 | 9.95 | 7.66 | 7.66 | 11.39 |
| hsa_circ_0003731 | | PTGR1 | 11.26 | | 11.18 | 11.74 | 11.65 | 10.78 | 9.95 | 7.66 | 10.19 | 10.01 |
| **upregulation (15)** | |  |  | |  |  |  |  |  |  |  |  |
| hsa_circ_0005719 | | NPHP4 | 7.66 | | 7.66 | 9.98 | 10.73 | 9.94 | 7.66 | 10.18 | 7.66 | 10.01 |
| hsa_circ_0007905 | | STX6 | 9.89 | | 7.66 | 11.35 | 10.73 | 11.70 | 9.95 | 11.97 | 7.66 | 11.39 |
| hsa_circ_0015839 | | CAMSAP2 | 7.66 | | 7.66 | 7.66 | 9.90 | 7.66 | 9.95 | 7.66 | 10.19 | 10.86 |
| hsa_circ_0017521 | | AKR1E2 | 7.66 | | 11.18 | 7.66 | 11.26 | 9.94 | 9.95 | 10.18 | 11.06 | 7.66 |
| hsa_circ_0021089 | | STK33 | 7.66 | | 7.66 | 7.66 | 7.66 | 10.78 | 9.95 | 10.18 | 10.19 | 7.66 |
| hsa_circ_0000441 | | MED13L | 9.89 | | 7.66 | 7.66 | 9.90 | 9.94 | 7.66 | 11.04 | 7.66 | 10.01 |
| hsa_circ_0101041 | | DNAJC3 | 9.89 | | 7.66 | 7.66 | 9.90 | 10.78 | 7.66 | 10.18 | 7.66 | 10.01 |
| hsa_circ_0000603 | | TEX9 | 9.89 | | 7.66 | 7.66 | 9.90 | 9.94 | 7.66 | 11.97 | 7.66 | 10.01 |
| hsa_circ_0035796 | | HERC1 | 7.66 | | 9.83 | 7.66 | 7.66 | 10.78 | 9.95 | 7.66 | 10.19 | 10.01 |
| hsa_circ_0006509 | | MYO9A | 7.66 | | 7.66 | 9.98 | 9.90 | 10.78 | 9.95 | 11.04 | 11.06 | 7.66 |
| hsa_circ_0000638 | | ETFA | 10.73 | | 7.66 | 7.66 | 9.90 | 11.70 | 9.95 | 7.66 | 11.60 | 10.01 |
| hsa_circ_0106383 | | SPECC1 | 7.66 | | 10.66 | 7.66 | 10.73 | 10.78 | 11.33 | 10.18 | 11.60 | 10.86 |
| hsa_circ_0007766 | | ERBB2 | 7.66 | | 10.66 | 7.66 | 11.26 | 9.94 | 7.66 | 11.04 | 10.19 | 10.86 |
| hsa_circ_0044396 | | IGF2BP1 | 7.66 | | 7.66 | 10.82 | 9.90 | 10.78 | 9.95 | 10.18 | 7.66 | 10.86 |
| hsa_circ_0000976 | | HPCAL1 | 7.66 | | 7.66 | 11.35 | 10.73 | 11.70 | 7.66 | 7.66 | 11.60 | 10.01 |

**Table S1.** The top 30 dysregulated circRNAs in A549 cells treated with different doses of radiation.
