# Supplementary material for: CircNEIL3 mediates pyroptosis to influence lung adenocarcinoma radiotherapy by upregulating PIF1 through miR-1184 inhibition
Source: Cell Death Dis. 2022 Feb 21;13(2):167. doi: 10.1038/s41419-022-04561-x (PMC8861163; doi:10.1038/s41419-022-04561-x)
Supplement: Supplementary file 4 — Table S2. The top 10 meaningful circRNAs based on the junction reads and logCPM values by edgeR. [file 41419_2022_4561_MOESM4_ESM.docx]

**Table S2.** The top 10 meaningful circRNAs based on the junction reads and logCPM by edgeR.

|  | **junction reads** | | | | | | | | | **logCPM by edgeR** | | | | | | | | |
| --- | --- | --- | --- | --- | --- | --- | --- | --- | --- | --- | --- | --- | --- | --- | --- | --- | --- | --- |
| **circRNA** | **0Gy-1/2/3** | | | **2Gy-1/2/3** | | | **4Gy-1/2/3** | | | **0Gy-1/2/3** | | | **2Gy-1/2/3** | | | **4Gy-1/2/3** | | |
| hsa_circ_0006156 | 3 | 1 | 2 | 2 | 1 | 1 | 0 | 1 | 0 | 11.26 | 9.83 | 10.82 | 10.73 | 9.94 | 9.95 | 7.66 | 10.19 | 7.66 |
| hsa_circ_0085616 | 3 | 1 | 2 | 2 | 0 | 1 | 0 | 1 | 0 | 11.26 | 9.83 | 10.82 | 10.73 | 7.66 | 9.95 | 7.66 | 10.19 | 7.66 |
| hsa_circ_0008797 | 3 | 1 | 2 | 1 | 0 | 1 | 0 | 1 | 0 | 11.26 | 9.83 | 10.82 | 9.90 | 7.66 | 9.95 | 7.66 | 10.19 | 7.66 |
| hsa_circ_0001346 | 12 | 8 | 4 | 15 | 4 | 5 | 2 | 2 | 6 | 13.17 | 12.52 | 11.74 | 13.49 | 11.70 | 12.02 | 11.04 | 11.06 | 12.34 |
| hsa_circ_0003731 | 3 | 3 | 4 | 4 | 2 | 1 | 0 | 1 | 1 | 11.26 | 11.18 | 11.74 | 11.65 | 10.78 | 9.95 | 7.66 | 10.19 | 10.01 |
| hsa_circ_0001460 | 7 | 4 | 3 | 1 | 1 | 2 | 0 | 0 | 1 | 12.41 | 11.56 | 11.35 | 9.90 | 9.94 | 10.80 | 7.66 | 7.66 | 10.01 |
| hsa_circ_0005982 | 4 | 2 | 6 | 1 | 2 | 1 | 0 | 0 | 3 | 11.64 | 10.66 | 12.30 | 9.90 | 10.78 | 9.95 | 7.66 | 7.66 | 11.39 |
| hsa_circ_0001772 | 3 | 1 | 4 | 3 | 2 | 0 | 1 | 3 | 1 | 11.26 | 9.83 | 11.74 | 11.26 | 10.78 | 7.66 | 10.18 | 11.60 | 10.01 |
| hsa_circ_0002457 | 0 | 1 | 1 | 2 | 1 | 0 | 2 | 1 | 3 | 7.66 | 9.83 | 9.98 | 10.73 | 9.94 | 7.66 | 11.04 | 10.19 | 11.39 |
| hsa_circ_0001523 | 2 | 0 | 0 | 0 | 1 | 0 | 1 | 1 | 6 | 10.73 | 7.66 | 7.66 | 7.66 | 9.94 | 7.66 | 10.18 | 10.19 | 12.34 |
