# Supplementary material for: CircNEIL3 mediates pyroptosis to influence lung adenocarcinoma radiotherapy by upregulating PIF1 through miR-1184 inhibition
Source: Cell Death Dis. 2022 Feb 21;13(2):167. doi: 10.1038/s41419-022-04561-x (PMC8861163; doi:10.1038/s41419-022-04561-x)
Supplement: Supplementary file 5 — Table S3. Primer sequences for RT-qPCR used in this study. [file 41419_2022_4561_MOESM5_ESM.docx]

**Table S3.** Primer sequences for RT-qPCR used in this study.

| **Gene** | **Forward primer, 5’ to 3’** | **Reverse primer, 5’ to 3’** | **Variation tendency** |
| --- | --- | --- | --- |
| hsa_circ_0006156 | GCAAGAAGCAGCCCAAAGTCG | TTGGAGACATGGCTGAGGGG | down-regulated |
| hsa_circ_0085616 | GGACTACAACTCGCCCACCA | GAGATCTGGTCCGGCATCCA | down-regulated |
| hsa_circ_0008797 | GCGAGACACACCTGCACTCT | TCCACACCACTATCCCCTGGA | down-regulated |
| hsa_circ_0001346 | AGTGGGCATCTGTCTCATCTTGA | GTGTGGCTGACAGCATGAGC | down-regulated |
| hsa_circ_0003731 | TGAAGAAAGCGTCTCCTGATGGT | GTCTGGCCACTCTGTCAGCA | down-regulated |
| hsa_circ_0001460 | GATGGGGAAAACAAGGGCAG | TTCCAAAAGTATTACACGGGT | down-regulated |
| hsa_circ_0005982 | TGTGCTCTTGGTTCAAGCTGG | TGGGGTCAAGGTAAGCAGCT | down-regulated |
| hsa_circ_0001772 | ACAGTATGAAGGCCACGAAGCT | GCCAGATAGCAAATCTTCTCCAAGTA | down-regulated |
| hsa_circ_0002457 | ACAGAATCCAGTTCGGGGCC | ACTGAGGCAGTCCTTTGTTACTGT | up-regulated |
| hsa_circ_0001523 | TCTTGGGCCCTGTGAACCTG | GGCGCTGGCACTGTAAACAG | up-regulated |
| GAPDH | GGCCTCCAAGGAGTAAGACC | AGGGGAGATTCAGTGTGGTG |  |
| U6 | CTCGCTTCGGCAGCACA | AACGCTTCACGAATTTGCGT |  |
| NEIL3 | GCAGGCTGCTGCACTGAATA | CCCAAAGTTTCCACGCCACT |  |
| PIF1 | GGGGCGAGATGGGATTGTG | ACCGATAAGTTTTTCACCAGCAT |  |
| β-actin | CCTGGCACCCAGCACAAT | GGGCCGGACTCGTCATAC |  |
