# Supplementary figures and images for: CircNEIL3 mediates pyroptosis to influence lung adenocarcinoma radiotherapy by upregulating PIF1 through miR-1184 inhibition
Source: Cell Death Dis. 2022 Feb 21;13(2):167. doi: 10.1038/s41419-022-04561-x (PMC8861163; doi:10.1038/s41419-022-04561-x)

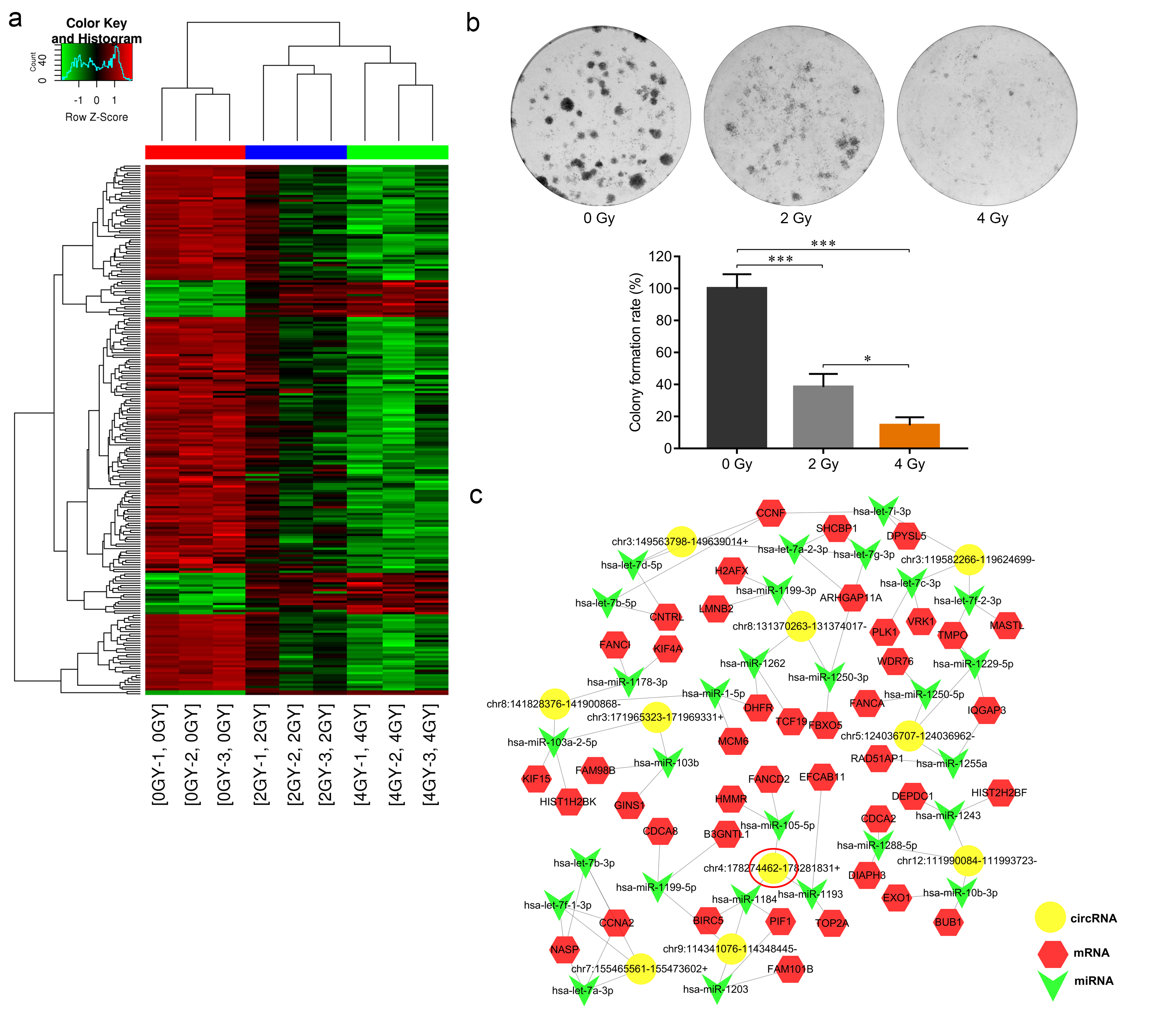

Supplement: Supplementary file 6 — Figure S1. Bioinformatics analysis of circRNAs in LUAD cells. [file 41419_2022_4561_MOESM6_ESM.tif]

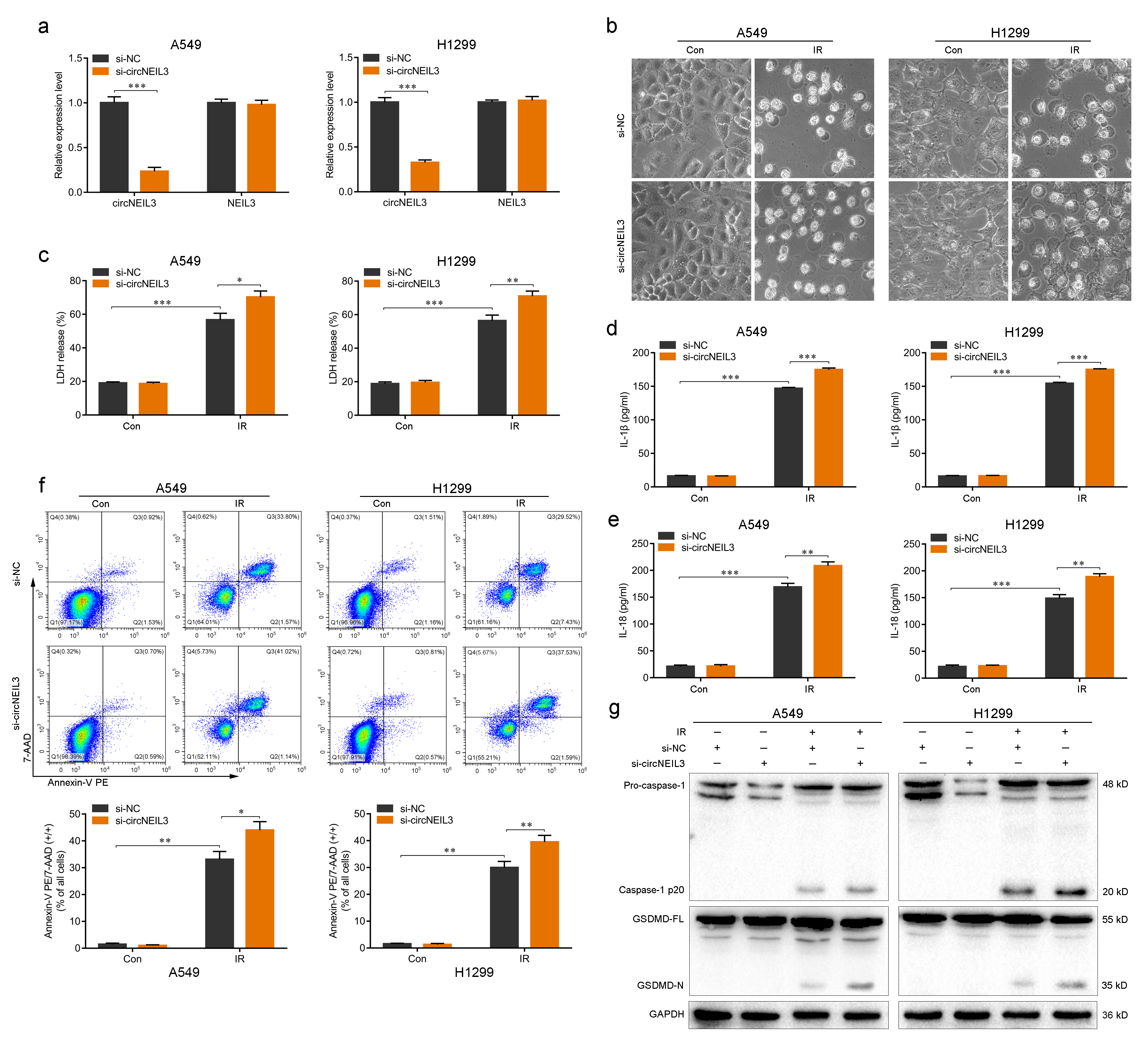

Supplement: Supplementary file 7 — Figure S2. circNEIL3 knockdown can promote irradiation-induced pyroptosis in LUAD cells. [file 41419_2022_4561_MOESM7_ESM.tif]

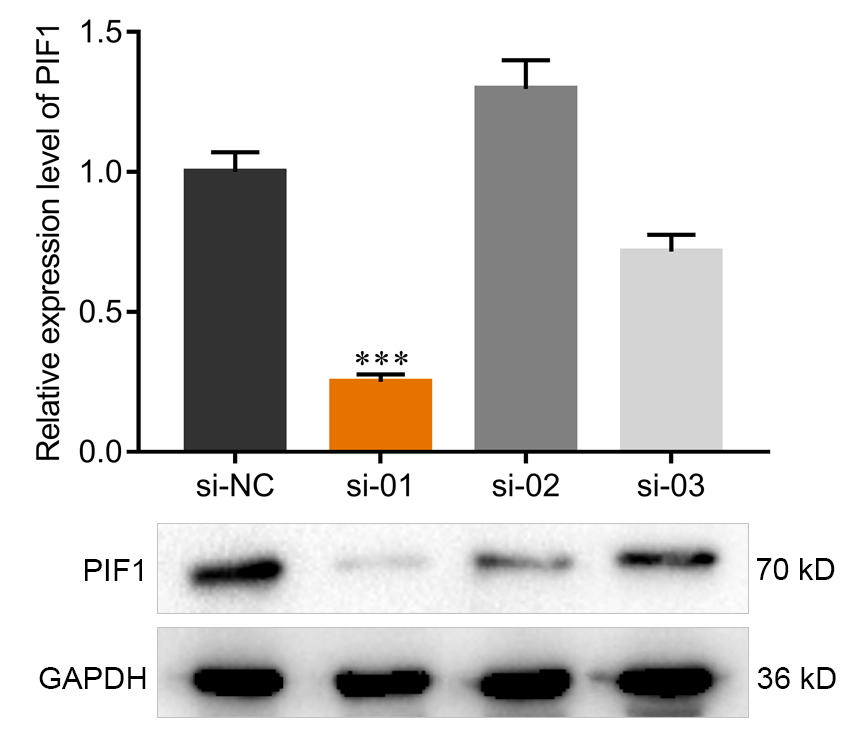

Supplement: Supplementary file 8 — Figure S3. Knockdown efficiency of PIF1 detected by RT-qPCR and western blot. [file 41419_2022_4561_MOESM8_ESM.tif]
